# Social Deficits and Repetitive Behaviors Are Improved by Early Postnatal Low-Dose VPA Intervention in a Novel *shank3*-Deficient Zebrafish Model

**DOI:** 10.3389/fnins.2021.682054

**Published:** 2021-09-10

**Authors:** Chunxue Liu, Yi Wang, Jingxin Deng, Jia Lin, Chunchun Hu, Qiang Li, Xiu Xu

**Affiliations:** ^1^Department of Child Health Care, Children’s Hospital of Fudan University, National Children’s Medical Center, Shanghai, China; ^2^Center for Translational Medicine, Institute of Pediatrics, Shanghai Key Laboratory of Birth Defects Prevention and Control, Children’s Hospital of Fudan University, National Children’s Medical Center, Shanghai, China

**Keywords:** *shank3*, valproic acid, zebrafish model, drug treatment, autism spectrum disorder

## Abstract

Mutations of the *SHANK3* gene are found in some autism spectrum disorder (ASD) patients, and animal models harboring *SHANK3* mutations exhibit a variety of ASD-like behaviors, presenting a unique opportunity to explore the underlying neuropathological mechanisms and potential pharmacological treatments. The histone deacetylase (HDAC) valproic acid (VPA) has demonstrated neuroprotective and neuroregenerative properties, suggesting possible therapeutic utility for ASD. Therefore, *SHANK3*-associated ASD-like symptoms present a convenient model to evaluate the potential benefits, therapeutic window, and optimal dose of VPA. We constructed a novel *shank3*-deficient (*shank3ab^–/–^*) zebrafish model through CRISPR/Cas9 editing and conducted comprehensive morphological and neurobehavioral evaluations, including of core ASD-like behaviors, as well as molecular analyses of synaptic proteins expression levels. Furthermore, different VPA doses and treatment durations were examined for effects on ASD-like phenotypes. Compared to wild types (WTs), *shank3ab^–/–^* zebrafish exhibited greater developmental mortality, more frequent abnormal tail bending, pervasive developmental delay, impaired social preference, repetitive swimming behaviors, and generally reduced locomotor activity. The expression levels of synaptic proteins were also dramatically reduced in *shank3ab^–/–^* zebrafish. These ASD-like behaviors were attenuated by low-dose (5 μM) VPA administered from 4 to 8 days post-fertilization (dpf), and the effects persisted to adulthood. In addition, the observed underexpression of *grm5*, encoding glutamate metabotropic receptor 5, was significantly improved in VPA-treated *shank3ab^–/–^* zebrafish. We report for the first time that low-dose VPA administered after neural tube closure has lasting beneficial effects on the social deficits and repetitive behavioral patterns in *shank3*-deficient ASD model zebrafish. These findings provide a promising strategy for ASD clinical drug development.

## Introduction

Autism spectrum disorder (ASD) encompasses a group of neurodevelopmental syndromes characterized by deficits in social interaction and communication as well as repetitive behaviors and restricted interests (American Psychiatric Association, 2013). There is strong evidence for the involvement of inherited genetic factors in ASD (accounting for at least 80% of the variation in disease risk) (Jutla et al., 2019; Liu et al., 2021). Furthermore, mutations in numerous genes encoding synaptic proteins have been identified in patients with ASD and intellectual disability (Verpelli and Sala, 2012; Lai et al., 2014). According to a meta-analysis, monogenic mutations in *SHANK3*, which encodes the major postsynaptic density (PSD) scaffolding protein at excitatory glutamatergic synapses, are found in approximately 0.69% of ASD cases and up to 2.12% of all moderate to profound intellectual disability cases (Leblond et al., 2014). *De novo* mutations, interstitial deletions, and terminal deletions have been identified in ASD (Durand et al., 2007; Moessner et al., 2007; Gauthier et al., 2009; Boccuto et al., 2013; Leblond et al., 2014). Additionally, *SHANK3* mutations underlie Phelan–McDermid syndrome (PMS, also known as 22q13.3 deletion syndrome), a rare autosomal dominant neurodevelopmental disorder characterized by autistic-like behaviors, absent to severely delayed speech, developmental delay, and moderate to profound intellectual disability as well as neonatal hypotonia and minor dysmorphic facial features (Phelan et al., 1993; Wilson et al., 2003; Phelan, 2008; Bonaglia et al., 2011; Phelan and McDermid, 2012). The genomic rearrangements in PMS are diverse, ranging from simple 22q13 deletions (72%), ring chromosomes (14%), and unbalanced translocations (7%) to interstitial deletions (9%), all leading to *SHANK3* haploinsufficiency (Bonaglia et al., 2011). Although the severity of the developmental delay tends to vary with deletion size (Sarasua et al., 2011; Zwanenburg et al., 2016), individuals with the same size deletion may exhibit vastly different degrees of disability (Dhar et al., 2010). Thus, *SHANK3* deficits appear to profoundly disrupt the neural circuitry required for social behavior, communication, and cognition.

The *SHANK3* gene (also known as ProSAP2, at 22q13.33) is the best studied of the three *SHANK* family members, which encoding an extensive number of mRNA and protein isoforms *via* multiple intragenic promoters and alternative splicing (Durand et al., 2007; Wang et al., 2014b). In the brain, *Shank3* mRNA is enriched in the cortex, thalamus, striatum, hippocampus, dentate gyrus, and cerebellar granule cells (Peca et al., 2011; Wang et al., 2016; Monteiro and Feng, 2017), suggesting important functions in synaptic plasticity underlying cortical organization, sensory processing, behavioral control, and cognition.

Owing to the strong genetic association between *SHANK3* deficiency and ASD, many studies have focused on the neurodevelopmental functions of this particular gene. Numerous animal models of *SHANK3* deficiency, including zebrafish, *Drosophila*, rat, mouse, and monkey models, demonstrate ASD-like behaviors, suggesting a causative role of *SHANK3* deficiency in ASD (Peca et al., 2011; Wang et al., 2016; Monteiro and Feng, 2017). Wang et al. (2016) reported that homozygous *Shank3* knockout mice displayed core behavioral features of ASD as well as impaired mGluR5–Homer association at the PSD, resulting in corticostriatal circuit abnormalities that may underlie learning deficits and ASD-like behaviors. In addition, monkey models also displayed core ASD features including impaired social interactions, repetitive behaviors, delayed vocalization, and reduced brain network activities (Tu et al., 2019; Zhou et al., 2019). The zebrafish genome harbors two homologs of human *SHANK3*, *shank3a*, and *shank3b*. In our previous studies, we generated the first *shank3b* loss-of-function mutation in zebrafish and reported prominent ASD-like behaviors (Liu et al., 2018). However, we did not examine the effects of *shank3a* and *shank3b* double mutant combinations, which would be more analogous to mammalian models harboring a single *SHANK3* mutation, or assess potential pharmacological strategies to mitigate behavioral deficits.

Current treatment options for ASD are limited, especially pharmacotherapies (Wang et al., 2014a; Penagarikano et al., 2015). Evidence-based treatments for ASD children are restricted mainly to educational practices, and intensive behavioral interventions such as Treatment and Education of Autistic and Related Communication-Handicapped Children (TEACCH) and the Early Start Denver Model (ESDM) (Dawson et al., 2010; Hyman et al., 2020). Outcomes of these behavioral therapies vary markedly according to intervention intensity, disease severity, and a variety of other factors (Sandbank et al., 2020). Further, education and behavioral interventions do not target the underlying neurobiological mechanisms (Wang et al., 2014a; Weitlauf et al., 2014) and are costly both for educational institutions and primary caregivers (Lord et al., 2018, 2020). Similarly, current pharmacologic treatments address only the associated symptoms or comorbidities, including agitation and hyperactivity, rather than the core symptoms and underlying causes (Fung et al., 2016; Lord et al., 2018, 2020; Muhle et al., 2018). Risperidone and aripiprazole are approved by the United States Food and Drug Administration (FDA) to treat comorbidities common in ASD, including irritability, and agitation (Lord et al., 2018, 2020), but similar to behavioral interventions, these evidence-based pharmacologic treatments lack sufficient biological support.

Based on available evidence, behavioral interventions should be implemented as early and intensively as possible following ASD diagnosis to improve the cognitive and adaptive outcomes of preschoolers (Weitlauf et al., 2014; Muhle et al., 2018). The preschool years are critical for acquiring language and social skills, key areas of difficulty in ASD, as this period coincides with the temporal window of enhanced plasticity in relevant neural circuits (Franz and Dawson, 2019). Similar to early intervention, pharmaceutical treatments appear effective in animal models when administered early, and Muhle et al. (2018) even suggested greater emphasis on early drug treatment rather than strict adherence to the standard timeline of efficacy based on studies in adults and adolescents. For instance, early postnatal treatment improves social deficits in adult mice with mutations in the ASD risk gene *cntnap2* (Franz and Dawson, 2019). Pharmacological inhibitors of histone deacetylase (HDAC) have garnered interest as possible ASD therapeutics due to demonstrated neuroprotective efficacy (Fischer et al., 2010). The class I HDAC inhibitor valproic acid (VPA) was found to reduce repetitive behaviors in a small randomized controlled trial involving 13 ASD children (Hollander et al., 2006). In addition, several studies have reported that VPA can attenuate irritability in young ASD patients (Hellings et al., 2005; DeFilippis and Wagner, 2016). Further, three daily VPA treatments transiently restored social preference deficits in adult *Shank3*-deficient mice, although the effect disappeared within a few days following treatment (Qin et al., 2018). Therefore, the effects of VPA on *shank3* mutant models warrant further study.

Here we investigated the developmental characteristics of *shank3*-deficient zebrafish, neurobehavioral features relevant to ASD, and the effects of various VPA treatment regimens. We speculated that VPA administration in the early postnatal period would be more effective at reversing the core ASD-like deficits in *shank3*-deficient zebrafish than juvenile or adult treatment.

## Materials and Methods

### Zebrafish and Embryo Maintenance

Wild-type (WT) zebrafish of Tu strains were acquired from Children’s Hospital of Fudan University. They were raised and maintained under standard laboratory conditions at 28.5°C in “system water” under a 14 h light/10 h dark cycle according to standard protocols (Kalueff et al., 2014; Evans and Erickson, 2019). Freshly fertilized eggs were collected from multiple breeding tanks containing 25 females and 25 males. All animal experimental procedures were in compliance with local and international regulations, and approved by the institutional animal care committee of Children’s Hospital of Fudan University.

### Generation of *shank3a* and *shank3b* Double Deficient Zebrafish Model

Zebrafish *shank3a* and *shank3b* genes and their exon/intron boundaries were identified by searching the NCBI database (gene ID: *shank3b*, NC_007115.7; *shank3a*, NC_007129.7). Mutations in *shank3a* and *shank3b* were generated using CRISPR/Cas9 editing as previously reported (Hwang et al., 2013; Mali et al., 2013). The CRISPR/Cas9 target of *shank3a* was 5′-GGACCCCAGCCCTCCTCCCGTGG-3′ and that of *shank3b* was 5′-GGGCGTGTTGTTGCCACGGCCGG-3′ (Liu et al., 2018; Supplementary Table 1). *In vitro*-transcribed RNA of the guide RNA (120 ng each) and Cas9 mRNA (500 pg) were microinjected into WT zebrafish embryos (*F*_0_) at the one-cell stage. The progeny were propagated *via* a series of out-crossings with WT zebrafish and genotyping of each generation. Eventually, these animals were in-crossed to obtain the homozygous knockouts *shank3a*^–/^*^–^* and *shank3b^–/–^*. The *shank3ab^–/–^* homozygous line was obtained by mutant crossing and subsequent genotyping.

### Behavior Tests for Adult Zebrafish

All behavioral experiments were conducted on 2–3.5 month old male zebrafish between 10 a.m. and 4 p.m. Behaviors were recorded for 30 min after 1–2 min habituation period using a video camera (zebrabox) suspended above the test tank. Zebrafish were returned to their home tanks immediately after completion of the test. The raw data was analyzed using Viewpoint software.

#### Open Field Test

The free-swimming open field test was performed in novel tanks as previously described (Liu et al., 2018). Each tank was 30 cm × 30 cm × 30 cm, and its walls consisted of opaque partitions. Swim velocity was calculated as the total distance traveled divided by the total swim time.

For the danger awareness test, the tank was virtually divided into two equal areas, peripheral and center, and greater peripheral swimming distance relative to central swimming distance was measured as a metric of danger awareness.

For analyzing repetitive and stereotyped behaviors, we used a double-blind method to score the swimming pattern within each minute, and counted the number of the four stereotyped swimming pattern episodes separately.

#### Shoaling Test

The shoaling test assesses social cohesion in homogeneous groups of zebrafish (Figure 3A; Liu et al., 2018). A shoal refers to a loose aggregation of individuals who swim close to one another, whereas a school describes a group of fish exhibiting polarized, synchronized motion (Perathoner et al., 2016). The distance between each fish can reveal the degree of shoaling behavior (i.e., social cohesion). Six zebrafish were placed in a novel 30 cm × 30 cm × 30 cm tank with walls consisting of opaque partitions, and mean inter-individual distance was measured (Kim et al., 2017).

#### Social Preference Test

Sociability was evaluated as the difference score between the time spent in proximity to a conspecific sector and an empty sector (Busnelli et al., 2016). Briefly, social testing was conducted over 30 min in a standard mating tank of inner dimensions 21 cm × 10 cm × 7.5 cm. A transparent plexiglas divider was placed in the middle of the tank, which allowed sufficient visual presentation for forming a social preference, and a single *shank3* mutant or WT zebrafish was placed on the left side while a group of six conspecific zebrafish (conspecific sector) was placed in the right side (Figure 3C). Social preference behavior was quantified as a distance distribution or as presence in a zone adjacent to the group of conspecifics. The distance ratio was calculated as the distance swam in the conspecific sector divided by the total distance.

#### Kin Preference Test

Another test was performed to assess preference for kinship using various colored variants. The duration and frequency of contact was compared between conspecifics and a phenotypically distinct strain (Figure 3E) in mating tanks with dimensions and configuration the same as those used in the “social preference test.” Briefly, two transparent separators divided the tank into three compartments, with a single test fish placed in the middle and Kin zebrafish placed on the right and non-Kin (red color) zebrafish placed on the left. Kin preference was represented by the ratio of the time spent in the Kin-sector to the total time.

### VPA Treatment and Phenotypic Assessments

To assess the extent to which VPA exposure affects morphology, 4 dpf WT or *shank3ab^–/–^* larvae were reared in Petri dishes containing blue egg water alone or blue egg water containing 5, 10, 20, or 50 μM sodium valproate (Cat No. 4543-10G, Sigma-Aldrich). The egg water with or without VPA was changed daily. At 8 dpf, larvae were observed under a microscope for mortality and any morphologic abnormalities, including distended abdominal and thoracic regions, lordosis, yolk sac edema, and pericardial edema. Adverse effects including mortality and malformation rates were calculated to determine the optimal VPA concentration for subsequent experiments (Supplementary Figure 1).

To examine the effects of early postnatal low-dose VPA exposure on autism-like behaviors, WT or *shank3ab^–/–^* larvae were exposed to blue egg water with or without 5 μM VPA from 4 to 8 dpf. At 8 dpf, each larva was pipetted into fresh paramecium liquid, and raised to 2.5 months old (juvenile) or 3.5 months old (adult). The juveniles were then examined for 30 min using the 1 versus 6 social preference assay, while adults were subjected to social preference, repetitive behavior, locomotor activity, and thigmotaxis tests to comprehensively evaluate the effects of VPA on autism-like behaviors.

### Real-Time Quantitative PCR

**FIGURE 1:**
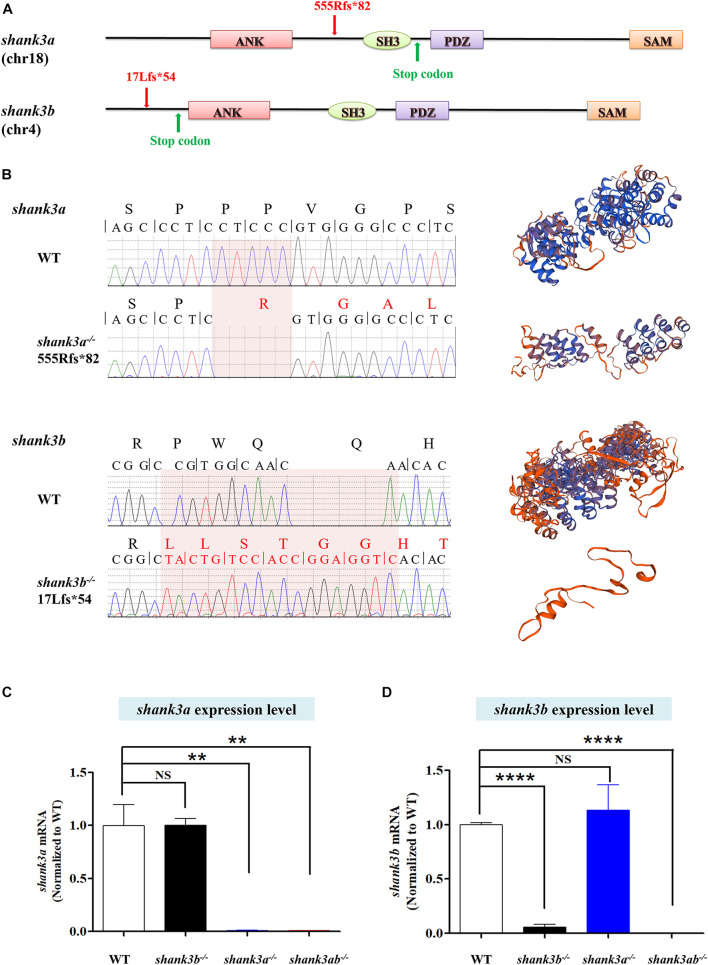
Generation of *shank3a* and *shank3ab* mutants in zebrafish by CRISPR-Cas9 gene editing. **(A)** Structures of zebrafish *shank3a* and *shank3b* (Liu et al., 2018) gene and protein domains (ANK, ankyrin repeat domain; SH3, Src homology 3 domain; PDZ, PSD-95/discs large/ZO-1 domain; SAM, sterile alpha motif domain). Exon 9 is the target for CRISPR/Cas9 gene editing in zebrafish *shank3a* and exon 2 is the target for *shank3b.* The CRISPR/Cas9 induced frameshift mutations in shank3a (5-base deletion) and in *shank3b* (5-base deletion and 13-base insertion) which led to the truncation of the protein (Liu et al., 2018). **(B)** The mutations of *shank3a* and *shank3b* (Liu et al., 2018) were verified by Sanger sequencing. The predictions of protein spatial structures were both suggested that shank3a and shank3b proteins were likely to turn into truncated proteins (https://www.swissmodel.expasy.org/interactive/wUdwQQ/models/). **(C)** Reduced expression level of *shank3a* mRNA in the brain of *shank3a^– /–^* and *shank3ab^– /–^* adult (4 mpf) male zebrafish analyzed by RT-qPCR, while *shank3b^– /–^* was not affected. **(D)** Reduced expression level of *shank3b* mRNA in the brain of *shank3b^– /–^* and *shank3ab^– /–^* adult (4 mpf) male zebrafish analyzed by RT-qPCR, while *shank3a^– /–^* was not affected. Data are shown as mean ± SEM; **P < 0.01. ****P < 0.0001.

Total RNA was extracted from 15 WT, *shank3a^–/–^*, *shank3b^–/–^*, and *shank3ab^–/–^* larvae each at 3.5–4.5 months post-fertilization (mpf) using the RNA Extraction Kit from Takara, and reverse transcribed to cDNA using the PrimeScript^TM^ reagent Kit with gDNA Eraser (Takara) according to the manufacturer’s recommendations. The Cas9 target region of *shank3a* and *shank3b* were amplified in duplicate samples from *shank3ab^–/–^* zebrafish by real-time quantitative PCR (RT-qPCR) to confirm genotype (Figures 1C,D and Supplementary Table 1).

To assess the effect of VPA exposure on synaptic genes and class I *hdac* genes (as VPA belongs to class I HDAC inhibitor), groups of ∼15 WT and *shank3ab^–/–^* zebrafish larvae were exposed to vehicle or VPA from 4 to 8 dpf, reared under normal conditions, then sacrificed for whole-brain total RNA isolation. The expression levels of the following genes analyzed by RT-qPCR: NMDAR subunits (*grin1a*, *grin1b*, *grin2bb*, *grin2ca*, *grin2da*, *grin2aa*), AMPAR subunits (*gria1a*, *gria1b*, *gria2b*), mGluR subunits (*grm1a*, *grm1b*, *grm5a*), and class I *hdac*s (*hdac1*, *hdac3*, *hdac8*). We selected β*-actin* or *Rpl13*α as internal controls because both are expressed in the brain throughout development. Primer sequence are shown in Supplementary Table 1.

### Western Blotting

Proteins (50 μg per sample) were separated by SDS-PAGE and transferred on polyvinylidene difluoride membranes. After blocking the membrane at room temperature for 2 h with 5% BSA or 7% skim milk, and incubated with primary antibodies at 4°C overnight at the following concentrations: NeuN (Abcam, ab177487, 1:1500), homer1 (Aviva systems biology, ARP40181_P050, 1:1000), and synaptophysin (Abcam, ab32594, 1:1500). The blots were washed in TBS containing 0.1% Tween-20 (TBST) and incubated with HRP-conjugated secondary antibodies (1:5000) for 1.5 h at room temperature. Following six washes in TBST, the blots were incubated with ECL reagent (BeyoECL Plus, P0018M) and exposed to Kodak X-ray film (Tanon 5200). The gray values of proteins were analyzed by ImageJ software (NIH, Bethesda, MD, United States^1^), and normalized to that of corresponding internal controls, β-actin (1:2000) or Vinculin (1:5000).

### Statistical Analysis

Values are presented as mean ± SEM. All data were analyzed using SPSS 20.0. In all experiments, WT and *shank3*-deficient zebrafish were compared by two-sided unpaired Student’s *t*-tests, while three or more groups were compared by analysis of variance (ANOVA). Genotypes within treatment groups were compared by one-way ANOVA. All experiments were conducted in triplicate using independently treated animals. A *P* < 0.05 was considered statistically significant for all tests.

## Results

### Generation of *shank3a^–/–^* and *shank3ab^–/–^* Zebrafish

To model *shank3* deficiency in zebrafish, we generated a loss-of-function mutant using CRISPR/Cas9 mutagenesis technology (Hwang et al., 2013; Mali et al., 2013; Liu et al., 2018). Generation of the *shank3b^–/–^* line was described in our previous study (Liu et al., 2018). Briefly, *shank3b^–/–^* harbors an early stop codon (p. 17Lfs^∗^54) due to a deletion of 5 bases and insertion of 13 bases, resulting in a frameshift mutation and a 90-amino acid truncated protein (Figure 1B). The *shank3a^–/–^* line carries a deletion of five bases leading to protein truncation (p. 555Rfs^∗^82), including most of the shank3 domain (Figure 1A). The *shank3ab^–/–^* line was generated by crossing *shank3b^–/–^* and *shank3a^–/–^*, and individuals were selected by genotyping. RT-qPCR confirmed that *shank3a* and s*hank3b* mRNA expression levels were significantly reduced in *shank3a^–/–^* and *shank3b^–/–^* zebrafish, respectively, and that both genes were downregulated in *shank3ab^–/–^* zebrafish (Figures 1C,D).

### Aberrant Morphology and Swimming Patterns of *shank3*-Deficient Zebrafish

**FIGURE 2:**
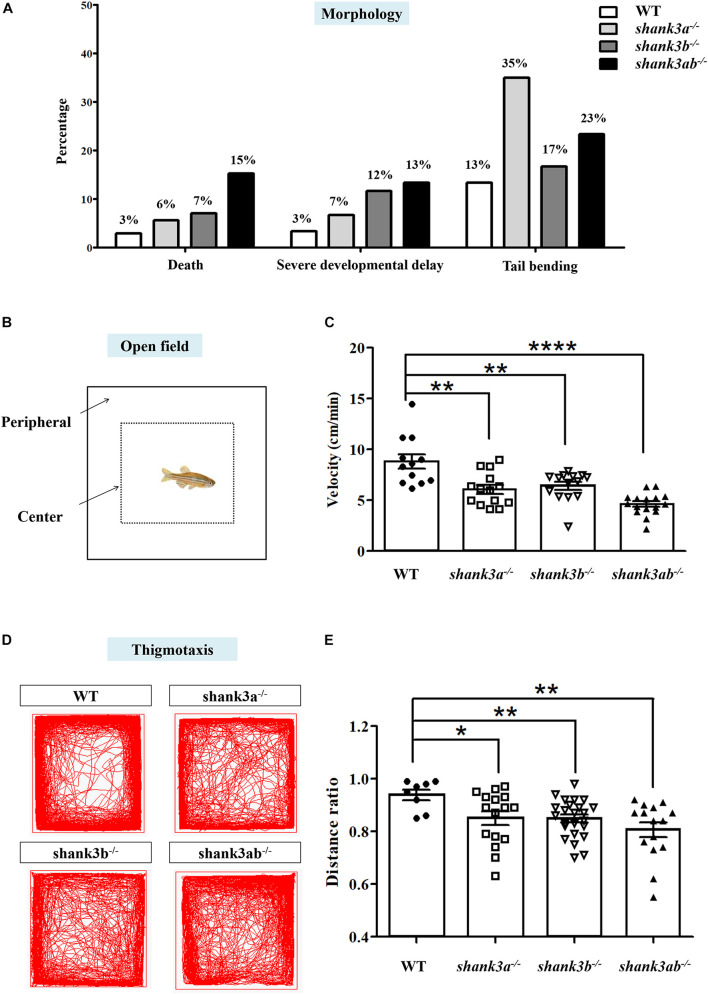
Morphological characteristics and locomotion activity alteration in *shank3*-deficient zebrafish. **(A)** Abnormal morphological changes in *shank3a^– /–^*, *shank3b^– /–^*, and *shank3ab^– /–^* larvae at ∼1 dpf, including death (WT, *N* = 340; *shank3a^– /–^*, *N* = 266; *shank3b^– /–^*, *N* = 270; *shank3ab^– /–^*, *N* = 118, severe developmental delay and tail bending (*N* = 60 each group). **(B)** Schematic diagram of the open field test and thigmotaxis test of adult male zebrafish (3.5 mpf). In the analysis of thigmotaxis test, the area of the peripheral zone is equal to the center zone (dotted line). **(C)** Compared with WT zebrafish (*N* = 12, 8.8 ± 2.4 cm/min), all *shank3*-deficient zebrafish displayed significantly decreased swimming velocity (*shank3a^– /–^*, *N* = 14, 6.1 ± 1.6 cm/min; *shank3b^– /–^*, *N* = 14, 6.4 ± 1.4 cm/min; *shank3ab^– /–^* zebrafish, *N* = 16, 4.6 ± 1.1 cm/min, respectively). **(D)** Representative traces of individual WT or *shank3*-deficient zebrafish in the thigmotaxis test. **(E)** Ratio for the distance traveled (periphery divided by the total zone) over 30 min in adult male zebrafish (3.5 mpf). WT, *N* = 8; *shank3a^– /–^*, *N* = 16; *shank3b^– /–^*, *N* = 24; *shank3ab^– /–^* zebrafish, *N* = 15. Data are shown as mean ± SEM; *P < 0.05, **P < 0.01, ***P < 0.001, ****P < 0.0001.

The developmental progression and morphological characteristics of *shank3* mutants were evaluated on 1 day post-fertilization (1 dpf). All mutants demonstrated a higher rate of mortality compared to WTs (*shank3a^–/–^*: 6%, 15/266; *shank3b^–/–^*: 7%, 19/270; *shank3ab^–/–^*: 15%, 18/118; WT: 3%, 10/340) (Figure 2A). In addition, the rate of severe developmental delay was significantly greater in the *shank3ab^–/–^* group compared to all other genotypes (*shank3ab^–/–^*: 13%, 8/60; *shank3a^–/–^*: 7%, 4/60; *shank3b^–/–^*: 12%, 7/60; WT: 3%, 2/60). Abnormal tail bending was more frequent in *shank3a^–/–^* zebrafish than other genotypes (*shank3a^–/–^*: 35%, 21/60; *shank3b^–/–^*: 17%, 10/60; *shank3ab^–/–^*: 23%, 14/60; WT: 13%, 7/60).

The remaining *shank3a^–/–^*, *shank3b^–/–^*, and *shank3ab^–/–^* zebrafish were viable and fertile into adulthood. The locomotor activities of adult *shank3a^–/–^*, *shank3b^–/–^*, and *shank3ab^–/–^* zebrafish were examined in the open home tank as previously described (Liu et al., 2018; Figure 2B). Compared to WT zebrafish, all *shank3*-deficient zebrafish displayed significantly decreased swimming velocity, with *shank3ab^–/–^* zebrafish demonstrating the slowest swimming speed (*shank3ab^–/–^*: 4.6 ± 1.1 cm/min; WT: 8.8 ± 2.4 cm/min; *shank3a^–/–^*: 6.1 ± 1.6 cm/min; *shank3b^–/–^*: 6.4 ± 1.4 cm/min) (Figure 2C).

Adult WT zebrafish typically avoid open areas near the water surface for protection against predation. To examine whether *shank3* deficiency modulates these avoidance behaviors, the relative proportions of swim time and distance in the pool periphery (thigmotaxis) versus the center (dotted line in Figure 2B) were calculated in a novel square tank with opaque walls. All *shank3*-deficient genotypes spent a significantly greater proportion of total swim time and traveled longer distances in the center of the tank compared to WT zebrafish, and *shank3ab^–/–^* zebrafish exhibited the greatest peripheral to center distance ratio of the three mutant genotypes (Figures 2D,E). This behavior can be interpreted as reduced alertness or reduced danger awareness (Mathur and Guo, 2010).

### Core ASD-Like Behaviors of *shank3*-Deficient Adult Zebrafish

**FIGURE 3:**
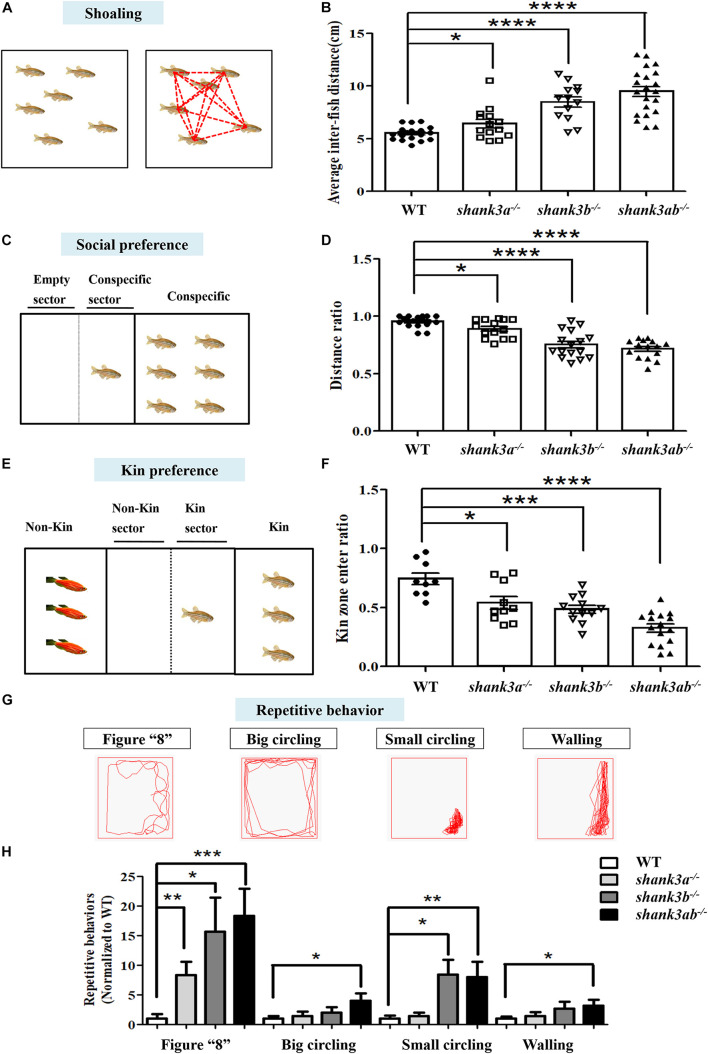
Core behavioral features of ASD-like displayed in *shank3*-deficient zebrafish. **(A,B)** The shoaling test showed significantly increased average inter-fish distance of adult male *shank3*-deficient zebrafish (3.5 mpf). WT, *N* = 18; *shank3a^– /–^*, *N* = 14; *shank3b^– /–^*, *N* = 13; *shank3ab^– /–^* zebrafish, *N* = 21. **(C,D)** The social preference test showed distance ratio in the conspecific sector were significantly reduced in *shank3*-deficient zebrafish compared to WT adult male zebrafish (3.5 mpf). WT, *N* = 16; *shank3a^– /–^*, *N* = 14; *shank3b^– /–^*, *N* = 16; *shank3ab^– /–^* zebrafish, *N* = 15. **(E,F)** The Kin recognition and preference test showed significantly reduced ratio of Kin zone entering in *shank3*-deficient zebrafish compared to WT adult male zebrafish (3.5 mpf). WT, *N* = 9; *shank3a^– /–^*, *N* = 10; *shank3b^– /–^*, *N* = 12; *shank3ab^– /–^* zebrafish, *N* = 16. **(G)** Representative trace of different types of stereotyped behaviors of *shank3*-deficient adult male zebrafish (3.5 mpf). **(H)** *shank3*-deficient zebrafish had a significantly higher proportion of stereotyped movements than WT zebrafish. WT, *N* = 12; *shank3a^– /–^*, *N* = 14; *shank3b^– /–^*, *N* = 14; *shank3ab^– /–^* zebrafish, *N* = 16. Data are presented as mean ± SEM; *P < 0.05, **P < 0.01, ***P < 0.001, ****P < 0.0001.

Since ASD diagnosis is based on behavioral criteria, a valid zebrafish model should exhibit core behavioral symptoms, including impaired social interactions and repetitive and stereotyped behaviors. The shoaling test showed that adult WT zebrafish spent the majority of swimming time in compact schools, while all three *shank3*-deficient genotypes swam in looser and larger schools with more frequent deviation (leaving the group), resulting in a greater average inter-fish distance compared to WT zebrafish (Figure 3B). This social deficit was particularly strong among the *shank3ab^–/–^* zebrafish group.

Social preference was further assessed by measuring conspecific proximity. WT zebrafish (3.5 mpf) maintain closer proximity with a conspecific group members on the right side (Figure 3C). In contrast, all *shank3*-deficient genotypes showed reduced frequency and duration of conspecific proximity, again with the *shank3ab^–/–^* genotype demonstrating the greatest average inter-conspecific distance (Figure 3D).

Wild type zebrafish also typically spend more time with a Kin group (conspecific and same color) than a non-Kin group in mixed populations. However, this Kin recognition and preference was markedly reduced in all *shank3*-deficient genotypes as measured by the proportion of time spent in close proximity with non-Kin (red-skinned) zebrafish among a mixed population (Figure 3F). Consistent with other social behavior tests, *shank3ab^–/–^* zebrafish spent the least amount of time in proximity to other conspecifics.

Repetitive and stereotyped behavior is another core symptom of ASD. Compared to adult WTs, *shank3ab^–/–^* zebrafish demonstrated greater behavioral perseveration, including repetitive stereotypic “figure 8” swimming, cycling behavior (swimming in circles), and other locomotor changes and patterns such as stereotyped “corner” or “wall” swimming (Figures 3G,H).

### Dysregulation of Synapse-Related Protein Expression in *shank3*-Deficient Zebrafish

**FIGURE 4:**
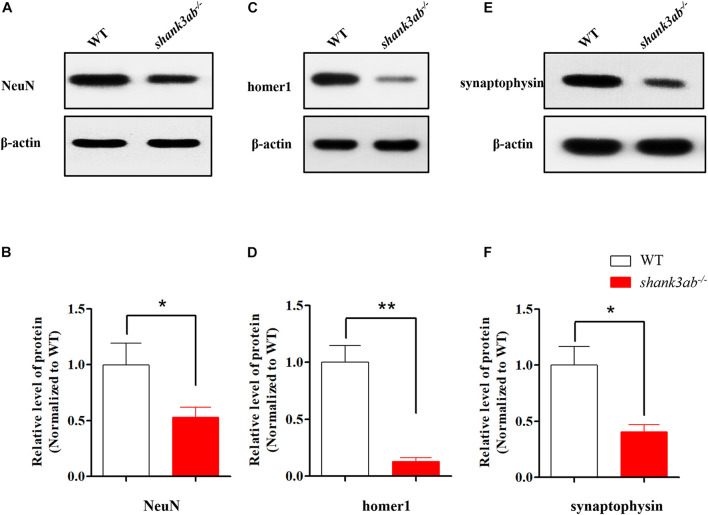
*shank3* deficiency resulted in the reduction of synapse-related proteins in adult zebrafish brain. **(A,B)** Quantitative immunoblot blot analysis showed that the neuron protein NeuN was significantly decreased (50% of WT) in the s*hank3ab^– /–^* male zebrafish brain relative to WT zebrafish (3.5 mpf). **(C,D)** The expression of post-synaptic homer1 protein was markedly reduced in s*hank3ab^– /–^* male zebrafish brain compared with that of WT zebrafish (3.5 mpf, 10% of WT). **(E,F)** The expression of presynaptic synaptophysin protein was significantly reduced in s*hank3ab^– /–^* male zebrafish brain compared with that of WT zebrafish (3.5 mpf, 36% of WT). *N* = 3 for each group. Data are presented as mean ± SEM; *P < 0.05, **P < 0.01.

These behavioral abnormalities in *shank3*-deficient zebrafish suggest possible disruption of normal synaptic function, so we compared the expression levels of several important synaptic proteins among genotypes. Expression of the neuronal marker NeuN was reduced by 50% in the brains of adult *shank3ab^–/–^* zebrafish compared to age-matched WTs (Figures 4A,B). As SHANK3 is a major synaptic scaffolding protein enriched at the PSD of excitatory synapses (Jiang and Ehlers, 2013; Monteiro and Feng, 2017), we also examined expression of the postsynaptic marker homer1 and found an approximately 90% reduction in *shank3ab^–/–^* zebrafish relative to WTs (Figures 4C,D). It was reported that *Shank3* deficiency in mice disrupts the presynaptic neurexin-neuroligin-mediated signaling pathway required for synapse targeting and development (Arons et al., 2012), so we further compared the expression levels of presynaptic proteins among genotypes, including the ubiquitous synaptic vesicle protein synaptophysin (Kwon and Chapman, 2011). Indeed, synaptophysin expression level was reduced by ∼64% in *shank3ab^–/–^* zebrafish compared to WTs (Figures 4E,F). Thus, shank3 deficiency reduced the expressions of several pre- and postsynaptic proteins which are likely important to protein transmitter signaling.

### Improved ASD Core Symptoms and *grm5* Receptor Expression by Early VPA Treatment

We then examined the efficacy of VPA to mitigate autism-like symptoms in these zebrafish models. In WT zebrafish, both *shank3a* and *shank3b* expression levels increased gradually from 3 to 7 dpf, a period of intense synaptogenesis (Liu et al., 2016). Exposure regimens of 3–7 and 4–8 dpf were thus judged as potentially suitable for optimal therapeutic effect. Based on preliminary observations, 4–8 dpf was chosen as the optimal exposure regimen (Supplementary Figure 1), and exposure concentrations (5, 20, and 50 μM) were then evaluated to identify the safety. We found that 5 μM completely eliminated mortality of *shank3ab^–/–^* larvae at 8 dpf and almost completely eliminated morphological dysgenesis (1.04%, 1/96) with no adverse effects on WT larvae (Supplementary Figure 1).

**FIGURE 5:**
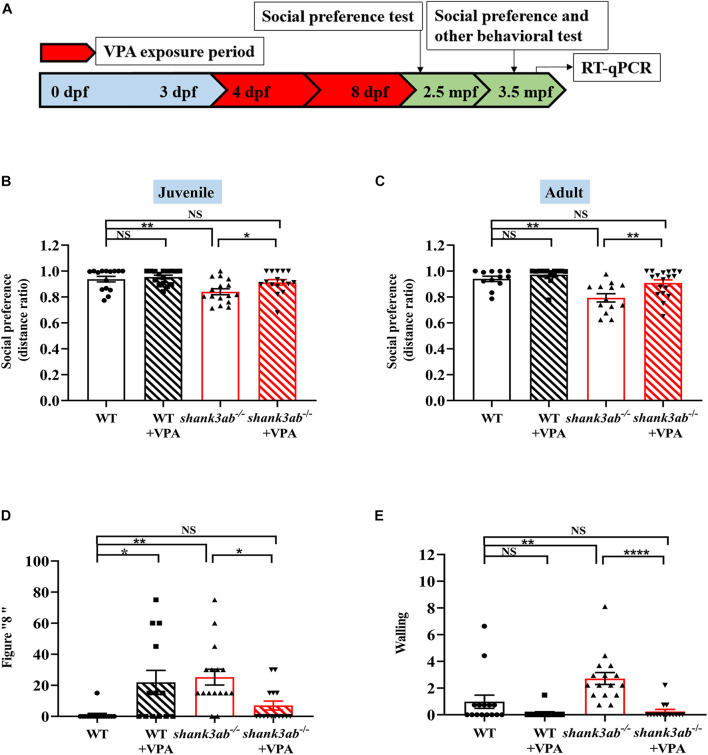
Improved ASD core symptoms in *shank3*-deficient zebrafish upon early VPA treatment. **(A)** Schematic overview of the protocol used for the VPA exposure period, the evaluation of behavioral tests at juvenile (2.5 mpf) and adult (3.5 mpf), and RT-qPCR analysis at 4.5 mpf. Red color indicates the VPA exposure phases. **(B)** Social preference index (distance ratio) of social test on juvenile zebrafish, WT, *n* = 15; WT-VPA, *n* = 16; *shank3ab^– /–^*, *n* = 16; *shank3ab^– /–^* -VPA, *n* = 16. **(C)** Social preference index (distance ratio) of social test on adult zebrafish, WT, *n* = 12; WT-VPA, *n* = 14; *shank3ab^– /–^*, *n* = 13; *shank3ab^– /–^* -VPA, *n* = 18. **(D)** The VPA treatment reduced abnormal proportion of stereotypic figure “8” swimming and **(E)** back and forth swimming (walling) in *shank3ab^– /–^* zebrafish, WT, *n* = 15; WT-VPA, *n* = 13; *shank3ab^– /–^*, *n* = 16; *shank3ab^– /–^* -VPA, *n* = 15. Data are shown as mean ± SEM. Statistical analyses: **(B)** one-way ANOVA with LSD correction for multiple testing. **(C–E)** One-way ANOVA with Bonferroni correction for multiple testing. Data are presented as mean ± SEM; *P < 0.05, **P < 0.01, ****P < 0.0001.

Once-daily administration of 5 μM VPA from 4 to 8 dpf (Figure 5A) significantly improved the social preference behavior of *shank3ab^–/–^* zebrafish both in juvenile and adulthood. In addition, VPA-treated *shank3ab^–/–^* juvenile (2.5 mpf) zebrafish spent significantly more time exploring a tank section containing conspecifics compared to an empty zone as measured by the distance ratio (*shank3ab^–/–^*: 0.84 ± 0.02; *shank3ab^–/–^* + VPA: 0.91 ± 0.02; *P* = 0.013) (Figure 5B). Moreover, VPA had no statistically significant effect on the social preference of WT zebrafish as measured by distance ratio (WT: 0.94 ± 0.02; WT + VPA: 0.95 ± 0.01; *P* = 0.558). This improvement was also observed in adult *shank3*-deficient zebrafish (3.5 mpf) (*shank3ab^–/–^*: 0.79 ± 0.03; *shank3ab^–/–^* + VPA: 0.91 ± 0.02; *P* = 0.006) (Figure 5C). In fact, the deficit in social preference relative to WTs was completely reversed by postnatal VPA (WT: 0.94 ± 0.02; *shank3ab^–/–^* + VPA: 0.91 ± 0.02).

Compared to untreated *shank3ab^–/–^* zebrafish, those receiving early postnatal VPA treatment also showed reduced frequencies of stereotypic “figure 8” swim patterns (*P* = 0.035) (Figure 5D) and “wall” swimming (*P* < 0.0001) (Figure 5E). Taken together, these results suggest that early low-dose VPA treatment can induce sustained reversal of core ASD-like symptoms in *shank3*-deficient zebrafish. Conversely, VPA-treated WT zebrafish showed a greater frequency of stereotypic “figure 8” swimming (*P* = 0.018) (Figure 5D). Moreover, postnatal VPA treatment rescued the deficient avoidance behavior of *shank3ab^–/–^* adult zebrafish as evidenced by a significant increase in peripheral to central distance ratio (*shank3ab^–/–^*: 0.77 ± 0.02; *shank3ab^–/–^* + VPA: 0.85 ± 0.02; *P* = 0.032; Supplementary Figure 2A). In contrast, VPA had no effect on the distance ratio of WT zebrafish (WT: 0.91 ± 0.02; WT + VPA: 0.88 ± 0.03) or the slower swim velocity of *shank3ab^–/–^* zebrafish (*shank3ab^–/–^*: 6.38 ± 0.20 cm/s; *shank3ab^–/–^* + VPA: 5.71 ± 0.25 cm/s; *P* = 0.890) (Supplementary Figure 2B).

**FIGURE 6:**
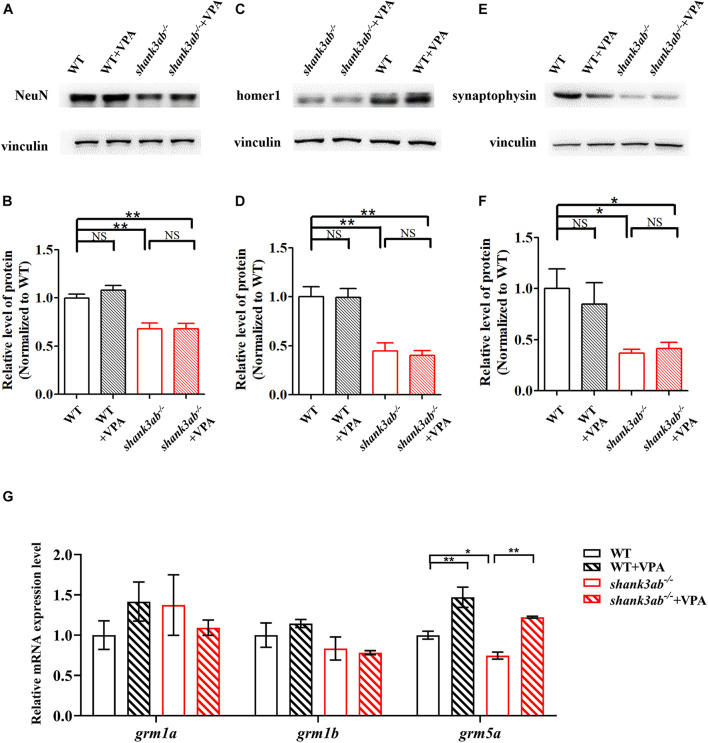
Increased *grm5* expression level in *shank3*-deficient zebrafish upon early VPA treatment. **(A,B)** Quantitative immunoblot blot analysis showed that the expression level of neuron protein NeuN was not significantly restored in the brain of s*hank3ab^– /–^* zebrafish treated with VPA relative to WT zebrafish (2 mpf). Similarly, the expressions of post-synaptic homer1 protein **(C,D)** and presynaptic synaptophysin protein **(E,F)** were not significantly increased in in the brain of s*hank3ab^– /–^* zebrafish treated with VPA relative to WT zebrafish (2 mpf). **(G)** The relative mRNA expression levels of *grm1a*, *grm1b*, and *grm5a* at 4.5 mpf were detected. Each group *n* = 3. Data are shown as mean ± SEM. *P < 0.05, **P < 0.01.

We also tested the effects of VPA treatment on synaptic proteins in *shank3ab^–/–^* zebrafish. As shown in Figures 6A–F, the expression levels of synaptic proteins (NeuN, homer1, and synaptophysin) were not significantly restored between WT and *shank3ab^–/–^* fish after exposed to VPA. To identify potential mechanisms underlying the amelioration of autism-like behaviors by early postnatal VPA, we first examined the expression levels of class I *hdac* genes (*hdac1*, *hdac2*, *hdac3*, and *hdac8*), as HDACs are the major known targets of this agent. Given the absence of *hdac2* gene in the zebrafish genome (Ko et al., 2019), we detected the expression levels of the rest three genes. However, RT-qPCR analysis revealed no changes in mRNA expression levels of *hdac1*, *hdac3*, and *hdac8* (Supplementary Figure 2C). We then examined the expression levels of glutamate receptors after cessation of treatment and found that VPA reversed the underexpression of *grm5a* observed in *shank3ab^–/–^* zebrafish (*P* < 0.05) (Figure 6G) but had no effects on the mRNA levels of AMPAR subunits (*gria1a*, *gria1b*, *gria2b*) (Supplementary Figure 2D), and NMDAR subunits (*grin1a*, *grin1b*, *grin2bb*, *grin2ca*, *grin2da*, *grin2aa*) (Supplementary Figure 2E) compared to untreated zebrafish.

## Discussion

We described a novel *shank3*-deficient zebrafish, *shank3ab^–/–^*, demonstrating stable autism-like behaviors from the juvenile stage through adulthood, including social deficits and stereotyped behaviors. These deficits were generally more severe than exhibited by either *shank3a* or *shank3b* mutants. All three mutants also exhibited higher postnatal mortality and rates of morphological dysgenesis than WTs, but adults were fertile. We also found there were decreases in several pre- and postsynaptic proteins in *shank3ab^–/–^* mutants. Low-dose VPA reversed some of these autism-like behaviors, consistent with the potential efficacy of this treatment strategy for ASD patients (Hellings et al., 2005; Hollander et al., 2006; DeFilippis and Wagner, 2016). Therefore, the *shank3ab^–/–^* zebrafish line is a robust model to explore the neurological mechanisms underlying ASD as well as potential pharmacological treatments.

Among molecular alterations, these *shank3*-deficient zebrafish exhibited a significant reduction in the expression levels of several synaptic proteins, including pre- and postsynaptic markers, which were consistent with previous mouse or *Drosophila* models. As a postsynaptic protein, change in postsynaptic homer1 protein was prominent in *shank3ab^–/–^* zebrafish, which was consistent with findings from other ASD mouse models (Tu et al., 1999; Wang et al., 2016). In addition, we also demonstrate reduced expression of the presynaptic protein synaptophysin, which was not previously detected in *Shank3* deficient mouse models. This finding suggests that *shank3* deficiency alters presynaptic formation and neurotransmission through direct or trans-synaptic mechanisms in zebrafish. The presynaptic functions of SHANK3 are not well characterized in comparison to postsynaptic functions. Several recent studies have suggested that SHANK3 is expressed in presynaptic terminals of rodent brain and dorsal root ganglion (DRG) neurons (Han et al., 2016). In a *Drosophila Shank* mutant (analogous to a *SHANK3* mutation in humans), Shank was found in both axons and the presynaptic sites of neuromuscular junctions (NMJs) (Wu et al., 2017). Moreover, ultrastructural analysis of synaptic boutons at *Shank* mutant calyces showed disorganization of both presynaptic and postsynaptic components, and lack of synaptic clefts (Wu et al., 2017). This first demonstration of reduced synaptophysin in a vertebrate ASD model supports a presynaptic function for shank3 protein. The contribution of shank3-associated presynaptic deficits to ASD warrant further investigation. Generally, the overall morphology of the brain tissues were relatively normal in KO group as compared to WT group (Supplementary Figure 3). Moreover, the expression levels of synaptic proteins were not significantly restored between WT and *shank3ab^–/–^* fish after exposed to VPA (Figure 6). Similarly, in mouse model, neuronal morphology and density were not changed between WT and *Shank3*-deficient mice and also not altered by romidepsin (a highly potent class I inhibitor) treatment (Qin et al., 2018). Additionally, there were no apparent morphological changes in neurons treated with VPA compared with controls (Fujiki et al., 2013).

Postnatal low-dose VPA treatment profoundly and persistently improved social preference deficits, abnormal repetitive behaviors, and impaired thigmotaxis, suggesting activation of a compensatory mechanism under *shank3* deficiency. Shank3 facilitates both synaptogenesis and the synaptic plasticity processes underlying social learning and cognition (Duffney et al., 2015). In addition, the behavioral abnormalities exhibited by *Shank3*-deficient animal models have been attributed to altered glutamatergic signaling (Peca et al., 2011; Wang et al., 2011; Bozdagi et al., 2013; Jiang and Ehlers, 2013), and VPA has been reported to increase synaptic transmission (Rinaldi et al., 2007; Akhtar et al., 2009).

We also found that postnatal low-dose VPA significantly reduced stereotyped swimming patterns (“figure 8” and “walling”). One possible explanation for this effect is improved transcription of *grm5a*, as Wang et al. (2016) demonstrated that disrupted mGluR5 scaffolding and abnormal mGluR5 signaling contribute to the excessive self-grooming and other behavioral and functional abnormalities of *Shank3*-defcient mice. Moreover, pharmacological enhancement of mGluR5 receptors rescued behavioral deficits, including repetitive behaviors and social deficits, in *Shank3* knockout mice (Vicidomini et al., 2017). Collectively, an important inference from this study is that early VPA treatment can have long-lasting benefits on repetitive behaviors in *shank3*-deficient zebrafish, possibly by improving *grm5a* expression.

Valproic acid has anticonvulsant and mood stabilizing activities and are used to treat epilepsy and bipolar disorder. Generally, VPA is a HDAC inhibitor (Phiel et al., 2001), a GABA transaminase inhibitor, and a sodium channel blocker (Johannessen, 2000; Löscher, 2002; Owens and Nemeroff, 2003; Zanatta et al., 2019). Several studies confirmed the role of HDAC inhibitor of VPA. Fujiki et al. (2013) have reported that VPA, trichostatin A and sodium butyrate (all are HDAC inhibitors), but not valpromide, which is a structural analog of VPA having the same antiepileptic effect as VPA but lacking the HDAC inhibitor activity, have proapoptotic effects on neural progenitor cells (NPCs) of embryonic stem (ES) cell-derived glutamatergic neurons. In this study, we also have added a positive control – romidepsin, a highly potent class I HDAC inhibitor, to treat fish (Supplementary Table 2 and Figure 4). WT or *shank3ab^–/–^* larvae were exposed to blue egg water with or without 0.05 or 0.1 μM romidepsin from 4 to 8 dpf. At 8 dpf, each larva was pipetted into fresh paramecium liquid, and raised to 2 months old (juvenile). Compared to *shank3ab^–/–^* zebrafish, romidepsin treated (0.05 or 0.1 μM) juvenile *shank3ab^–/–^* zebrafish exhibited significantly elevated social preference behaviors, which was consistent with *Shank3*-deficient mice model (Qin et al., 2018).

Valproic acid is a broad-spectrum inhibitor against class I (HDAC1, HDAC2, HDAC3, and HDAC8) HDAC (Chelladurai et al., 2020). Qin et al. (2018) reported that *Shank3*-deficient mice exhibited an abnormally low level of histone acetylation resulting from *HDAC2* upregulation in the prefrontal cortex (PFC) and β-catenin/HDAC2 played a causal role in social deficits of *Shank3*-deficiency mouse model and the therapeutic effect of romidepsin. While the levels of *HDAC1*, *HDAC3*, *and HDAC8* mRNA were largely unchanged. Given the absence of *hdac2* gene in the zebrafish genome (Ko et al., 2019), we detected the expression levels of the rest three genes. Similarly, RT-qPCR analysis revealed no changes in mRNA expression levels of *hdac1*, *hdac3*, and *hdac8* (Supplementary Figure 2C). In the further study, a VPA analog that does not have the HDAC inhibitory activity should be used as a control to detect the effects of the HDAC inhibitory activity of VPA.

This study also supports previous studies demonstrating that the early postnatal period is a critical therapeutic time window for long-lasting effects on core autistic symptoms (Dinstein et al., 2011). Hensch (2005) reported that novel interventions should be applied before irreversible neural function changes coinciding with the end of these critical periods. However, clinical studies have demonstrated that VPA exposure before the neural tube is closed (20–24 days of gestation in humans) increases the incidence of neurodevelopmental disorders including ASD (Rice and Barone, 2000; Jentink et al., 2010; Meador et al., 2013). Moreover, prenatal VPA exposure is actually used to establish rodent ASD models. Animals exposed to VPA during neural tube closure [E12.5 in rats according to Kim et al. (2011) and E10.5 in mice according to Kim et al. (2014)] showed an increased incidence of autism-like symptoms. When VPA was administered earlier than this critical time point, embryonic malformation was very likely to occur (Kim et al., 2019). In contrast, VPA administered after neural tube closure did not cause embryonic lethality or autism-like phenotypes (Kim et al., 2019). Furthermore, long-term VPA therapy had no noticeable noxious effect on cognition and learning in school children (Calandre et al., 1990). Thus, the optimal postnatal time window appears essential for VPA treatment efficacy against ASD.

Dose was the other key factor for effective VPA treatment. In animal models, fetal VPA exposure impairs cognitive outcome and increases malformation rate in a dose-dependent manner (Meador et al., 2013; Kaplan et al., 2015). Nicolini and Fahnestock (2018) reported ASD-like deficits following exposure of rat embryos to 350-600 mg/kg or of mouse embryos to 300-800 mg/kg VPA, while exposure to 25 μM from 10 to 24 hpf elicited social deficits in zebrafish (Baronio et al., 2018). VPA can also induce neuronal apoptosis (Ikonomidou et al., 1999). Conversely, low-dose VPA induced a dramatic rescue of core autistic deficits in *shank3*-deficient zebrafish, and this dose had no detectable effects on WT animals.

This study provides a new genetic zebrafish model of *shank3* deficiency which displayed distinctly abnormal social behaviors and increased stereotyped behaviors. Importantly, the autism-like behaviors could be improved by postnatal low-dose VPA treatment. These findings may suggest a path for further research to identify medicinal development and allow for more in-depth understandings of future clinical drug research.

## Data Availability Statement

The original contributions presented in the study are included in the article/Supplementary Material, further inquiries can be directed to the corresponding authors.

## Ethics Statement

The animal study was reviewed and approved by Research Ethics Board of Children’s Hospital of Fudan University.

## Author Contributions

XX and QL conceived the study. CL and YW performed the experiments, analyzed the data, and wrote the initial draft of the manuscript. All authors contributed to the interpretation of the results, provided critical feedback, helped shape the analysis and manuscript, and approved the submitted manuscript.

## Conflict of Interest

The authors declare that the research was conducted in the absence of any commercial or financial relationships that could be construed as a potential conflict of interest.

## Publisher’s Note

All claims expressed in this article are solely those of the authors and do not necessarily represent those of their affiliated organizations, or those of the publisher, the editors and the reviewers. Any product that may be evaluated in this article, or claim that may be made by its manufacturer, is not guaranteed or endorsed by the publisher.

## Footnotes

1
http://rsb.info.nih.gov/ij/
