# Supplementary material for: Social Deficits and Repetitive Behaviors Are Improved by Early Postnatal Low-Dose VPA Intervention in a Novel shank3-Deficient Zebrafish Model
Source: Front Neurosci. 2021 Sep 10;15:682054. doi: 10.3389/fnins.2021.682054 (PMC8462462; doi:10.3389/fnins.2021.682054)
Supplement: Supplementary file 1 [file Data_Sheet_1.pdf]

Supplementary materials

Social deficits and repetitive behaviors are improved by early postnatal low-dose VPA intervention in a novel *shank3*-deficient zebrafish model

This file contains Supplementary Table 1-2, Supplementary Figure 1-4 and Legends, and Supplementary methods.

Supplementary Tables and Figures

Supplementary Table S1. gRNA gene-target sequences, primers for PCR genotyping and RT-qPCR probes used in this study

Item	*Exon/Intron*	*Sequence (5'-3')*	*Amplicon length (bp)*	*Annealing temp (°C)*	*Ensemble gene ID*
**gRNA target: *shank3b***	ex2	GGGCGTGTTGTTGCCACGGCCGG			NC_007115.7
**PCR genotyping**	in1	ATATGGGTCTTGCTTGTATACAGGCGA	500	60	
	in2	GAATCAACAACTCGGTCTGTACCAATCA		60.3	
**gRNA target: *shank3a***	ex9	GGACCCCAGCCCTCCTCCCGTGG			NC_007129.7
**PCR genotyping**	in8	AAAGGCAGCCGTTAACGGGAG	493	59.2	
	in9	ACAGCTGTCAGAAGCAAATAGATAAGCAAA		60.1	
**RT-qPCR**					
shank3b	ex2	CGGCCGTGGCAACAACAC	245	59	XM_07355263.2
	ex3	TTAAGCACATCGGTCAGGCTTTGT		59	
shank3a	ex5/6	GGCACTTATTACGCTGCTGGATCTG	191	60	XM_021467755.1
	ex6/7	CATGACGGCAAGCCTGGTGAAT		60.7	
hdac1	ex5/6	AGCTGCTCAAGTACCATCAAAGA	201	52.7	NM_173236
	ex7	TGAGCGGGTAATTTACAGCG		53.1	
hdac3	ex7/8	CCCGGGAACTGGTGATATGTATGAAG	249	60.5	NM_200990
	ex9/10	ACAAACTCCACACATTCTCCATGTCC		59.1	

hdac8	ex8	GTGTCTAACCTATATACTTGGCTGGG	270	53.8	NM_213431
	ex10/11	ACATTCTTCAGATTCCCTTTGATGG		56.5	
gria1a (AMPAR subunit)	ex2/3	CATGACTTATGCGTTTTGTTCG	220	52.7	NM_205598
	ex3	CCACCTGTAGTGCTCGATTACA		52	
gria1b (AMPAR subunit)	ex6	TTGACAGTAAAGACTTGAAAAGCCC	218	55.1	NM_205730
	ex7/8	AAACTGCACCTGCTGTAAGGCT		54.8	
gria2b (AMPAR subunit)	ex5/6	AAACCTGGGTTTTGTGGATGG	236	55.1	NM_131895
	ex7	TCTGTTTGTGCAGGTAACGGAAA		54.8	
grin1a	ex3	ATTTCCAGTCAGGTGTACGCGA	204	55.7	NM_001076714
	ex9/10	TACGTGTGCCTGGTGGGAATAT		55	
grin1b	ex9/10	GATTGTAACAATTCATCAAGAGCCC	217	55	NM_001144131
	ex11	GGTGAAGTTCATTGTGAATGCAAGT		56.1	
grin2aa	ex3/4	TGCACAAGTACATGATGAACGTG	221	53.6	XM_021473854
	ex5	GGTCAGGTGATTGTCATCTGTCTC		54.3	
grin2bb	ex5/6	TACGAAAGGGTCGGTAAATGG	247	53.3	NM_001128337
	ex6/7	TGTAGATCCCTGAATCTCCTGTCA		54.3	
grin2ca	ex7/8	TGATCGGAGAGGTGTTTTATAAGC	204	53.6	NM_001365787
	ex9	TGACGAACATCATGACCCACA		54.3	
grin2da	ex3/4	TGAAGGAGAATGAAGCACAGGT	303	53	XM_009294079
	ex4	AATTCCCTGCGAAACTCGTC		53.3	
grm1a	ex1/2	TACATACAGAAGGCAACTATGGGG	260	54.1	XM_002665739
	ex2	CCCAATTAGCTGGAACTCTCCA		54.9	
grm1b	ex2/3	CGGTTCACACAGAGGGAAACTA	264	53.4	NM_001302252
	ex3	TGCCGATGAGGAGGAATTCT		53.6	
grm5a	ex2	AGCTTTGGGCAAGTGACTTCTC	164	54.3	NM_0013028710
	ex1/2	TTCACACTGAAGGGAATTATGGAG		54	

β-actin	ex2	CGAGCTGTCTTCCCATCCA	102	60	ENSDART00000055194
	ex3	TCACCAACGTAGCTGTCTTTCTG		60	
Rpl13α	ex4/5	TCTGGAGGACTGTAAGAGGTATGC	148	53.4	ENSDART00000023156
	ex6	AGACGCACAATCTTGAGAGCAG		53.8	

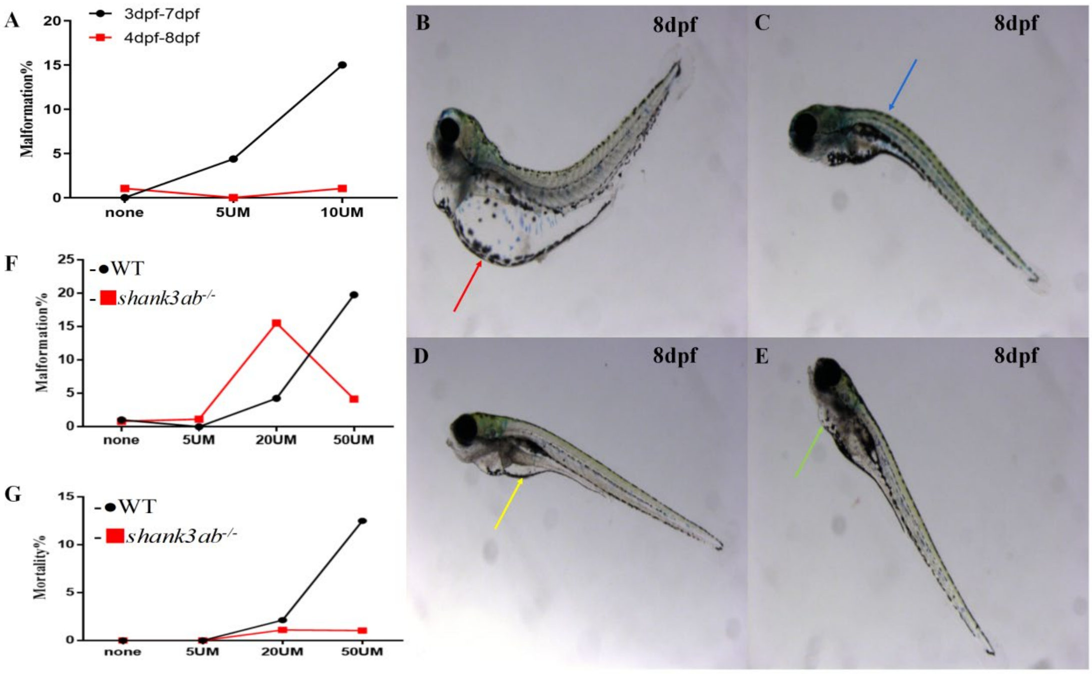

Supplementary Figure S1 The malformation and mortality of larva treated with various exposure concentrations VPA. (A) The malformation of WT larva treated with two different exposure paradigms (3 - 7 dpf and 4 - 8dpf) of VPA. 0 μM: 3 - 7 dpf (0%, 0/39), 4 - 8dpf (1.04%, 1/96). 5 μM: 3 - 7 dpf (4.35%, 2/46), 4 - 8dpf (0, 0/96). 10 μM: 3 - 7 dpf (15%, 6/40), 4 - 8dpf (1.04%, 1/96). The malformation including distended abdominal and thoracic regions, lordosis, yolk sac edema and pericardial edema. (B-G) The malformation and mortality of larva treated with various exposure concentrations VPA (5 μM, 20 μM and 50 μM) from 4 dpf to 8 dpf. (B) Distended abdominal and thoracic regions (red arrow). (C) Lordosis (blue arrow). (D) Yolk sac edema (yellow arrow). (E) Pericardial edema (green arrow). (F) The phenotypic deformities of larva treated with VPA, 0 μM-WT (1.04%, 1/96), 0 μM-*shank3ab*$^{-/-}$ (0.82%, 1/122), 5 μM-WT (0, 0/96), 5 μM-*shank3ab*$^{-/-}$ (1.14%, 1/88), 20 μM-WT (4.26%, 4/94), 20 μM-*shank3ab*$^{-/-}$ (15.56%, 14/90), 50 μM-WT (19.79%, 19/96), 50 μM-*shank3ab*$^{-/-}$ (4.17%, 4/96). (G) The mortality of larva treated with VPA, 0 μM-WT (0%, 0/96), 0 μM-*shank3ab*$^{-/-}$ (0%, 0/122), 5 μM-WT (0, 0/96), 5 μM-*shank3ab*$^{-/-}$ (0%, 0/88), 20 μM-WT (1.11%, 1/94), 20 μM-*shank3ab*$^{-/-}$ (2.13%, 2/90), 50 μM-WT (1.04%, 1/96), 50 μM-*shank3ab*$^{-/-}$ (12.5%, 12/96).

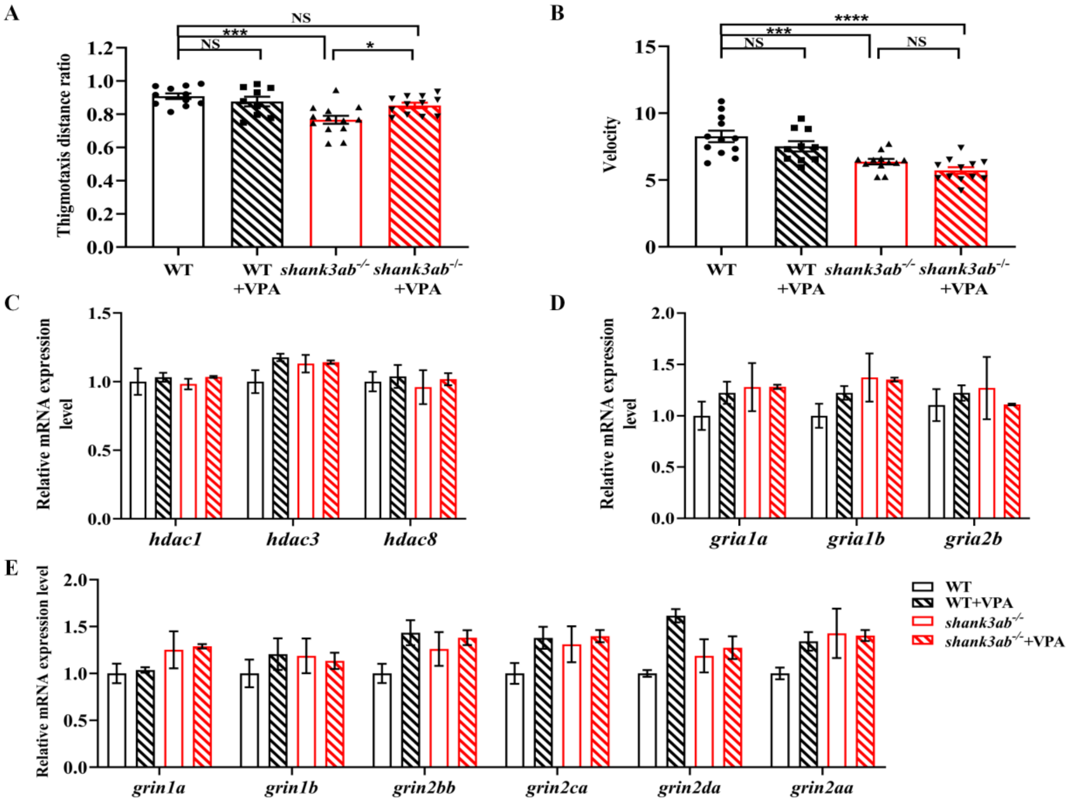

Supplementary Figure S2 The effects of fear/danger responses (A), velocity (B) on adult (3.5 mpf) *shank3ab*$^{-/-}$ zebrafish， and relative mRNA expression level of *hdac*s (C), *gria* (D) and *grin* (E) on adult (4.5 mpf) *shank3ab*$^{-/-}$ zebrafish under VPA exposure. (A) WT, n=11; WT-VPA, n=9; *shank3ab*$^{-/-}$, n=13; *shank3ab*$^{-/-}$-VPA, n=12. Data are shown as mean ± SEM. Statistical analyses: One-way ANOVA with Bonferroni correction for multiple testing. * $P < 0.05$, *** $P < 0.001$. (B) WT, n=12; WT-VPA, n=10; *shank3ab*$^{-/-}$, n=12; *shank3ab*$^{-/-}$-VPA, n =12. Data are shown as mean ± SEM. Statistical analyses: One-way ANOVA with Bonferroni correction for multiple testing. *** $P < 0.001$, **** $P < 0.0001$. (C-E) The relative mRNA expression level of associated genes at 4.5 mpf, normalized to WT. Each group n=3. Data are shown as mean ± SEM. Statistical analyses: One-way ANOVA with LSD correction for multiple testing.

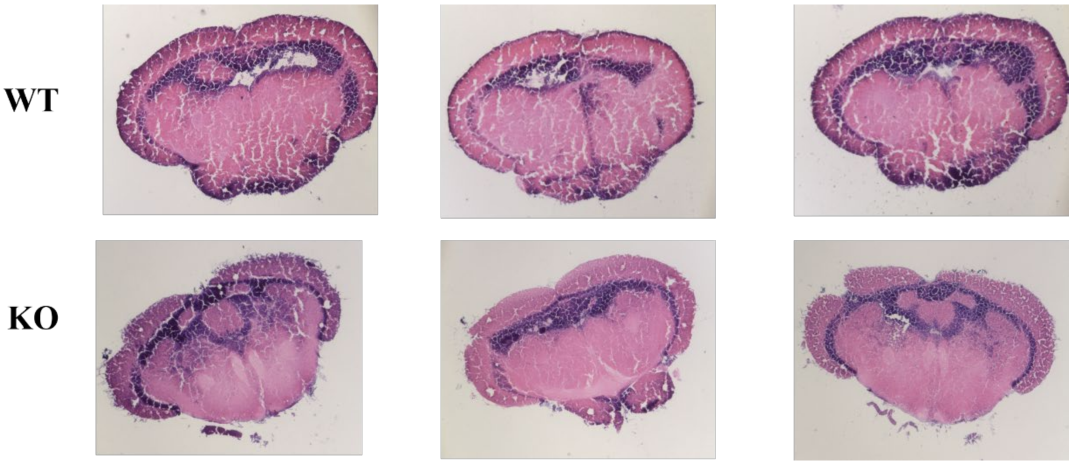

Supplementary Figure S3 Hematoxylin and eosin (HE) staining of zebrafish brain (4 mpf) between WT and shank3 KO group.

Supplementary Table S2. The malformation and mortality of larva treated with various exposure concentrations romidepsin (RMD)

	**WT**	**WT+0.1μM RMD**	**WT+0.05μM RMD**	***shank3ab***$^{-/-}$	***shank3ab***$^{-/-}$ **+0.1μM RMD**	***shank3ab***$^{-/-}$ **+0.05μM RMD**
**Mortality**	0%(0/81)	0%(0/76)	0%(0/79)	0%(0/18)	0%(0/39)	0%(0/38)
**Lordosis**	0%(0/81)	0%(0/76)	1.3%(1/79)	0%(0/18)	0%(0/39)	0%(0/38)
**Pericardial edema**	0%(0/81)	0%(0/76)	0%(0/41)	0%(0/18)	0%(0/39)	0%(0/38)

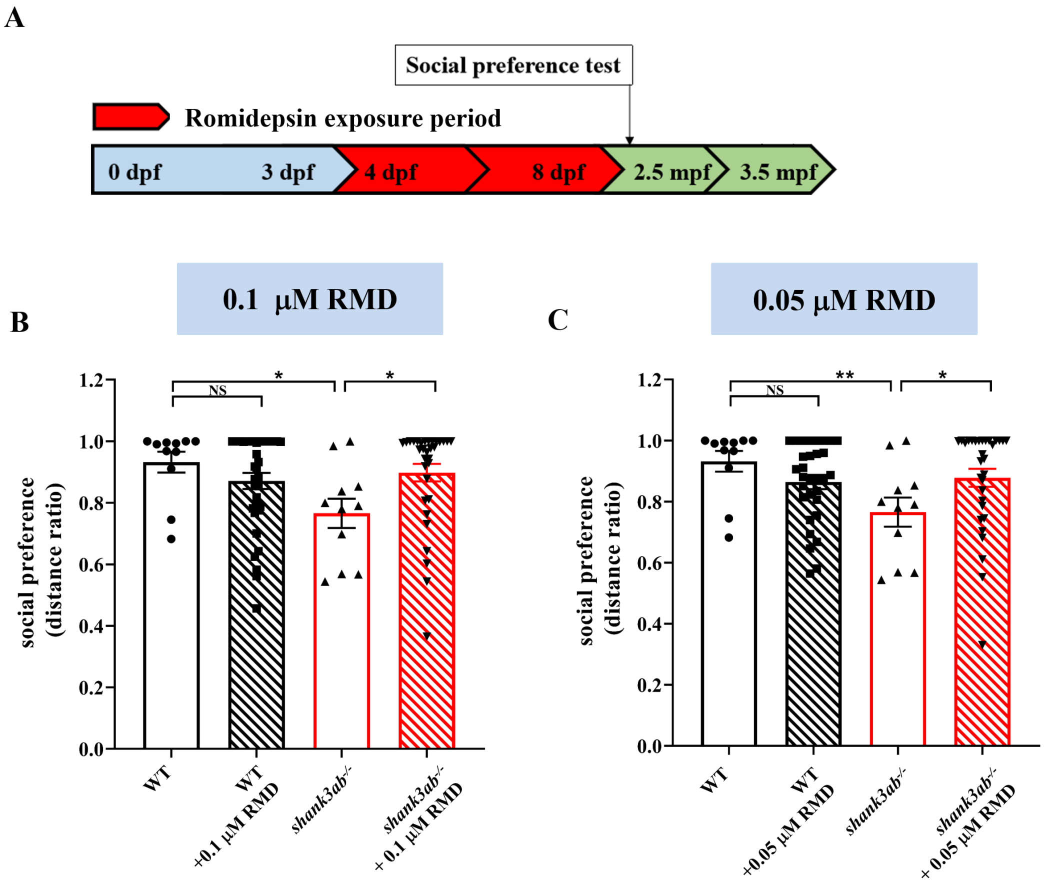

Supplementary Figure S4. Improved ASD core symptoms in *shank3*-deficient zebrafish upon romidepsin (RMD) treatment. (A) Schematic overview of the protocol used for the romidepsin exposure period, the evaluation of behavioral tests at juvenile (2 mpf). **(B)** Social preference index (distance ratio) of social test on juvenile zebrafish treated by 0.1 μM RMD, WT, n=11; WT-0.1 μM RMD, n=35; *shank3ab*$^{-/}$, n=11; *shank3ab*$^{-/-}$-0.1 μM RMD, n =32. **(C)** Social preference index (distance ratio) of social test on juvenile zebrafish treated by 0.05 μM RMD, WT, n=11; WT-0.05 μM RMD, n=31; *shank3ab*$^{-/}$, n=111; *shank3ab*$^{-/-}$-0.05 μM RMD, n =31. One-way ANOVA with LSD correction for multiple testing. Data are presented as mean ± SEM; * $P < 0.05$, ** $P < 0.01$.

Supplementary methods

Romidepisn administration

Romidepisn (Selleckcem) was prepared by dissolving it in DMSO to make stock solutions (100 μM) and diluting it with egg water before administration (DMSO concentration of the working solutions: 0.1% and 0.05%). Wild-type or *shank3ab*$^{-/-}$ larvae were exposed to blue egg water with or without 0.05 or 0.1 μM romidepsin from 4 dpf to 8 dpf. At 8 dpf, larvae were observed under a microscope for mortality and Lordosis, and then was pipetted into fresh paramecium liquid, and raised to 2 months old (juvenile). The juveniles were examined for 30 minutes using the 1 vs 6 social preference assay to evaluate the effects of romidepisn on autism-like behaviors.
